# The Effect of Type 2 Resistant Starch and Indole-3-Propionic Acid on Ameliorating High-Fat-Diet-Induced Hepatic Steatosis and Gut Dysbiosis

**DOI:** 10.3390/foods13111625

**Published:** 2024-05-23

**Authors:** Min Yang, Wanhao Cai, Xinxin Li, Yixuan Deng, Jinjun Li, Xin Wang, Liying Zhu, Chong Wang, Xiaoqiong Li

**Affiliations:** 1 Key Laboratory of Applied Technology on Green-Eco-Healthy Animal Husbandry of Zhejiang Province, College of Animal Science and Technology and College of Veterinary Medicine, Zhejiang Agriculture and Forestry University, Hangzhou 311300, China; 2020608022038@stu.zafu.edu.cn (M.Y.); 2023608031001@stu.zafu.edu.cn (W.C.); 2 State Key Laboratory for Managing Biotic and Chemical Threats to the Quality and Safety of Agro-Products & Institute of Food Sciences, Zhejiang Academy of Agricultural Sciences, Hangzhou 310021, China; lxx04011121@163.com (X.L.); lijinjun@zaas.ac.cn (J.L.); wangxin@zaas.ac.cn (X.W.); zhuliying@zaas.ac.cn (L.Z.); 3 The 2nd School of Medicine, Wenzhou Medical University, Chashan University Town, Wenzhou 325035, China; dengyixuan0711@163.com

**Keywords:** obesity, type 2 resistant starch, indole-3-propionic acid, high-fat diet, hepatic steatosis, gut dysbiosis

## Abstract

Owing to the interplay of genetic and environmental factors, obesity has emerged as a significant global public health concern. To gain enhanced control over obesity, we examined the effects of type 2 resistant starch (RS2) and its promoted microbial-derived metabolite, indole-3-propionic acid (IPA), on hepatic steatosis, antioxidant activity, and gut microbiota in obese mice. Neither RS2 nor low-dose IPA (20 mg kg^−1^) exhibited a reduction in body weight or improved glucose and lipid metabolism in post-obesity state mice continuously fed the high-fat diet (HFD). However, both interventions improved hepatic steatosis, with RS2 being more effective in all measured parameters, potentially due to changes in gut microbiota and metabolites not solely attributed to IPA. LC-MS/MS analysis revealed increased serum IPA levels in both RS2 and IPA groups, which positively correlated with *Bifidobacterium* and Clostridium. Moreover, RS2 exhibited a more significant restoration of gut dysbiosis by promoting the abundance of health-promoting bacteria including *Faecalibaculum* and *Bifidobacterium*. These findings suggest that the regulatory role of RS2 on tryptophan metabolism only partially explains its prebiotic activity. Future studies should consider increasing the dose of IPA and combining RS2 and IPA to explore their potential interventions in obesity.

## 1. Introduction

With social and lifestyle changes, obesity and its associated comorbidities, such as type 2 diabetes mellitus (T2DM) and non-alcoholic fatty liver disease (NAFLD), are becoming an epidemic [1]. More than four million people died in 2017 because they were overweight or obese. Of the changes in people’s lifestyles related to obesity nowadays, dietary changes are considered to be the biggest contributing factor [2]. Importantly, there are many data suggesting that the gut microbiota is implicated in the physiological outcomes of dietary changes [3,4].

The gut microbiota is essential for human well-being as it regulates a wide range of metabolic processes, including the breakdown of nutrients and the production of microbial metabolites that are vital components of the overall systemic metabolic system [5]. Serum indole-3-propionic acid (IPA) is correlated with the risk of T2DM and obesity, representing a microbial metabolite protective against non-alcoholic steatohepatitis (NASH) [6,7]. IPA, an indole metabolite derived from the gut, is linked to the consumption of dietary fiber [8]. *Clostridium* (C.) *caloritolerans*, *C. paraputrificum*, *C. cadvareris*, *Peptoniphilus* (P.) *asaccharolyticus*, *P. russellii*, *P. anaerobius*, and *P. stomatis* can utilize tryptophan to generate IPA [9]. Bacteria, such as *Lactobacillus reuteri* [10], *Akkermansia*, and the *Clostridium genus* [11], including C. *sporogenes* [9] and C. *caloritolerans* [12], might be associated with IPA production. It has been reported that IPA, like melatonin, scavenges free radicals and protects against oxidative damage [13]. IPA is commonly acknowledged to possess anti-inflammatory, anti-hyperglycemic, and neuroprotective effects, both in laboratory studies and animal models [14,15]. Although research on the function of IPA is increasing, studies on how to promote tryptophan metabolism for IPA production through diet so as to improve metabolism are currently still lacking.

Studies have shown that type 2 resistant starch (RS2) can significantly increase the concentration of IPA in mouse plasma and is positively correlated with an improvement in the abundance of *Allobaculum* and *Bifidobacterium* in the intestinal microbiota [16]. Thus, IPA may be a microbial metabolite through which RS2 induces health benefits [16]. Dietary fibers such as RS2 have well-established beneficial effects on metabolism [17,18]. Resistant starch decreases intrahepatic triglycerides in patients with NAFLD via gut microbiome alterations, and also has the ability to lose and improved insulin sensitivity [19,20]. The majority of dietary fibers cannot be indigestible in the intestine due to their unique structural and physical properties, so they are fermented into short-chain fatty acids (SCFAs) in the large intestine [21]. RS2 is a raw granule that has undergone numerous evaluations in many studies [22]. The FDA has granted approval to Hi-maize-resistant starch, a commercially available RS2 supplement derived from naturally modified high-amylose corn. This supplement is utilized in the treatment of patients diagnosed with T2DM [23]. In addition, it possesses prebiotic properties that facilitate the growth of beneficial bacteria in the gastrointestinal tract [24]. While the exact mechanisms responsible for these advantages are likely diverse, there is growing recognition that the gut microbiota plays a pivotal role in driving these positive effects on health [21]. Recent studies have shown that RS2 was redefined as a native starch granule containing nanocrystals with orthorhombic structure [25], thereby opening avenues for investigating how it promotes the growth of beneficial intestinal bacteria and regulates glucose and lipid metabolism.

RS2 is a probiotic widely recognized for its ability to promote gut microbiota metabolism of tryptophan to generate the anti-inflammatory metabolite IPA. The objective of this study is to compare the effects of RS2 and IPA on steatosis, antioxidant activity, and gut microbiota in diet-induced obese (DIO) mice. We also provide potential strategies to promote the development of functional foods for the treatment of related diseases.

## 2. Materials and Methods

### 2.1. Ethics Statement

All animal study protocols followed the guidelines of the Institutional Animal Care and Use Committee (IACUC) at the Zhejiang Academy of Agricultural Sciences, with ethics approval granted under the number ZAAS2020041.

### 2.2. Sample Preparation

High-amylose cornstarch (Hi-maize 260, RS2) containing 66.4% total dietary fiber was supplied by Ingredion Food Ingredients Co., Ltd. (Shanghai, China). Per 100 g of RS2, it includes 0.8 g of fat, 40.6 g of carbohydrates (all starch), 48.0 g of fiber, and 0.6 g of protein. Sigma-Aldrich was the supplier from which IPA was acquired. (St. Louis, MO, USA). IPA was dissolved in 0.1 M NaOH, and the pH was adjusted to 7.2 prior to use.

### 2.3. Animals and Experimental Protocols

C57BL/6 J mice were ordered from GemPharmatech Co., Ltd. (Nanjing, China). To generate diet-induced obesity models, an HFD (60% kcal/fat, D12492, Research) was introduced at 6 weeks of age and fed to them for 12 consecutive weeks before purchase. The mice were housed in a room with constant temperature conditions (25 ± 2 °C) and humidity (60 ± 5%), maintained on a 12 h light-dark cycle. And they had ad libitum access to food. Following a 10-day acclimatization period, mice in the negative control (CON) group were switched to a normal diet (MD12031) with a fat content of 10% kcal/fat. Additionally, they received 200 µL of water orally. The remaining DIO mice were still fed the HFD diet and were randomly assigned to three groups to receive the following three treatments (*n* ≥ 5): (1) HFD group: DIO mice received 200 µL water solution; (2) RS2 group: DIO mice received 2 g kg^−1^ body weight RS2 dissolved in 200 µL water via oral gavage; and (3) IPA group: DIO mice received 20 mg kg^−1^ body weight IPA via oral gavage. The doses of 2 g kg^−1^ day^−1^ RS2 and 20 mg kg^−1^ day^−1^ IPA were equivalent to 4.8 g RS2 and 97.6 mg IPA day^−1^ by a 60 kg human according to the Km factor ratio of 3 and 37 for mice (20 g) and humans (60 kg), respectively [26]. According to a previous report, oral IPA at a dose of 20 mg kg^−1^ day^−1^ alleviates HFD-induced liver injury and produces no adverse reactions [27]. At the end of week 12, fresh fecal samples were collected in individual sterile tubes and stored at −80 °C for subsequent microbiota analysis. Mice were fasted overnight prior to euthanasia. Fresh blood samples were then collected via abdominal aorta puncture immediately before euthanization, allowed to stand at room temperature for 30 min, and then followed by centrifugation at 3000 rpm for 20 min. The supernatant serum was collected, transferred into clean centrifuge tubes, and stored at −80 °C. After being killed by decapitation following CO_2_ stunning, mice liver tissues were collected and placed into sterile cryovials, then stored at −80 °C. Liver, fat, and pancreatic tissue sections were also collected, fixed in 4% paraformaldehyde, and prepared for further analysis.

### 2.4. Biochemical Analysis of the Liver Function

An automatic biochemical analyzer (Minday, Shenzhen, China) was used to detect the blood levels of triglycerides (TG), total cholesterol (TC), high-density lipoprotein (HDL-C), low-density lipoprotein (LDL-C), aspartate aminotransferase (AST), and alanine aminotransferase (ALT). An automatic Omron glucose analyzer (Omron, Dalian, China) was used to detect the blood levels of glucose (Glu).

### 2.5. Assessment of Oxidative Stress and Lipid Peroxidation

Using FastPrep-24^TM^ Tissue and cell homogenizer (MP Biomedicals, Santa Ana, CA, USA) ground into tissue homogenate, glutathione peroxidase (GSH-PX), malondialdehyde (MDA), catalase (CAT), glutathione (GSH), total antioxidant capacity (T-AOC), and superoxide (SOD) were assessed using an assay kit according to the manufacturer’s instructions (Nanjing Jiancheng Bioengineering Institute, Nanjing, China).

GSH-PX activity was determined via the colorimetric method. The MDA content was tested using the TBA method. The CAT content was tested using the visible light and molybdate amine methods. The GSH content was tested using the microplate method. The T-AOC content was determined using the ABTS method. The SOD content was tested using the WES-1 method.

### 2.6. Targeted Metabolomics

Since metabolites in the gut flora vary widely, only serum tryptophan metabolites were tested. An amount of 400 µL of methanol was added to 100 µL of collected serum, vortexed, and mixed; thereafter, the solution was centrifuged at 14,000 rpm for 10 min at 4 °C. After taking 450 μL of supernatant, we added 450 μL of methanol to an EP tube containing precipitate, vortexed and mixed until the precipitate was suspended in liquid, and ultrasonicated for 5 min until the precipitate was completely dissolved. The solution was subsequently centrifuged at 14,000 rpm for 10 min at 4 °C. Thereafter, 450 μL of supernatant was taken and vortexed. The solution was again centrifuged at 14,000 rpm for 10 min at 4 °C. The supernatant was subjected to liquid chromatography/tandem mass spectrometry (LC/MS-MS) analysis for the quantification of tryptophan metabolite levels in serum. LC-MS/MS analysis was conducted using a QTRAP^TM^ 6500 mass spectrometer (Sciex, Framingham, MA, USA) connected to an AQUITY UPLC system, which consisted of a thermostatic automatic sampler and an ultra-high-performance binary pump (I-class, Waters, MA, USA). Chromatographic separation was achieved on an ACQUITY PREMIER BEH C18 column (1.7 μm, 2.1 × 150 mm, 1/pk, Waters, Milford, DE, USA) at 45 °C. The control software used for the LC-MS system was Analyst 1.6.2.

### 2.7. Histological Staining

Adipose, liver, pancreas, ileum, and colon tissues were harvested and fixed in 4% paraformaldehyde. Subsequently, the tissues were embedded in paraffin following standard protocols to prepare sections. Hematoxylin and eosin (HE) staining was performed on the pancreatic sections, which were then examined under a light microscope for morphological analysis.

For the liver sections, they were fixed with 4% paraformaldehyde at room temperature for 1 h. After fixation, the sections were cryoprotected in 20% sucrose at 4 °C overnight and embedded in OCT. The resulting cryosections, with a thickness of 12 µm, were stained with Oil Red O.

### 2.8. DNA Extraction, Sequencing, and Data Analysis

Cecal contents were aseptically extruded from the cecum. The V3–V4 region of the bacterial 16S rRNA gene was amplified using the primers 338F (5′-ACTCCTACGGGAGGCAGCA-3′) and 806R (5′-GGACTACHVGGG TWTCTAAT-3′) on an ABI GeneAmp^®^ 9700 PCR thermocycler (ABI, Foster City, CA, USA). The resulting amplicons were purified and quantified using a DNA Gel Extraction Kit (Axygen Biosciences, Union City, CA, USA) and a QuantiFluor™-ST, respectively. Next-generation sequencing was carried out on an Illumina MiSeq platform following the standard protocols of Majorbio Bio-Pharm Technology Co., Ltd. The raw sequence data have been deposited in the NCBI Sequence Read Archive (SRA) database under the accession number PRJNA935523.

The paired-end (PE) reads obtained from MiSeq sequencing were split into individual samples. Quality control was performed on the reads based on sequencing quality, and the overlapping regions between the paired reads were used to generate optimized data after quality control and merging. The optimization data were further processed using the DADA2 denoising method to obtain representative sequences and abundance information for each amplicon sequence variant (ASV). The species annotation database used was Silva138/16s_bacteria, with a confidence threshold of 0.7 [28]. After removing the ASV with a sequence number of less than 0.1% in all the sequences and flattening according to the minimum number of sample sequences, the effective sequence of each sample was 23,785. Alpha and beta diversity analyses were performed using the R 3.3.1 software and vegan package (version 3.3.1). The linear discriminant analysis (LDA) effect size (LEfSe) method was used to identify the effect of each differentially abundant taxon. Additionally, redundancy analyses (RDA) and Spearman correlations were used to associate abundant differential taxa with tryptophan metabolites. A Wilcoxon rank-sum test and Welch’s *t*-test were used to compare the data, and the significance value was set to 0.05.

### 2.9. Statistical Analyses

Except for the bioinformatic information, statistical analyses were performed using SPSS software (version 12.0; IBM Corp., Armonk, NY, USA) and GraphPad Prism (version 9.0.0). Data are presented as mean ± SEM in the text. Significant differences (*p* < 0.05) between groups were evaluated using a one-way ANOVA. Significant differences in the figures are indicated by asterisks: *, **, and ***, corresponding to *p* < 0.05, <0.01, and <0.001, respectively.

## 3. Results

### 3.1. Effects of RS2 and IPA on Hepatic Steatosis in Obese Mice

**Figure 1:**
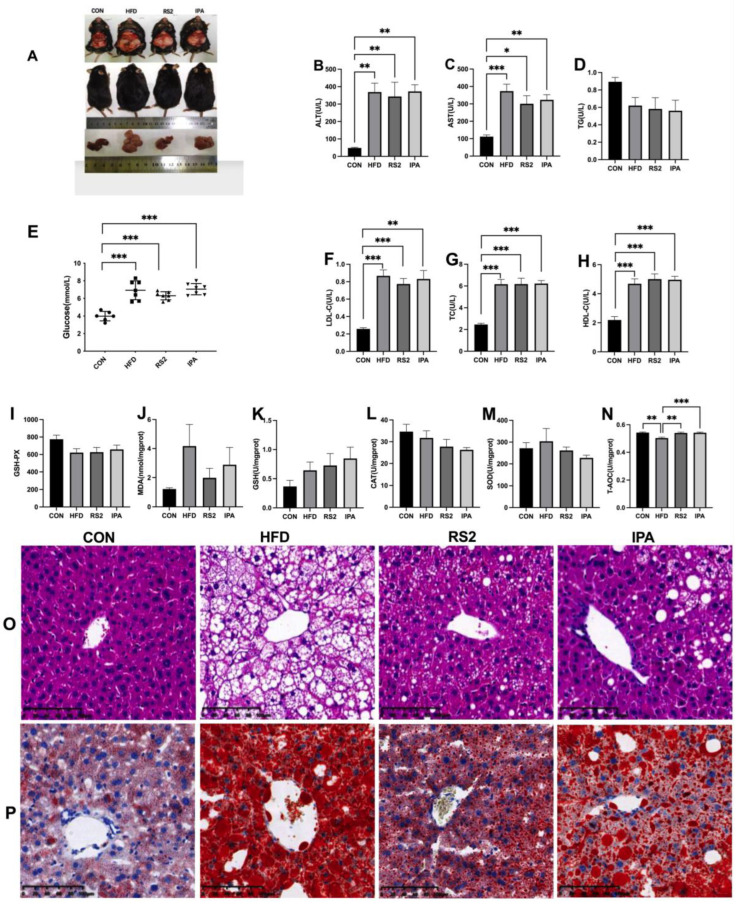
Representative mouse and liver images (**A**). Serum levels of alanine aminotransferase (ALT) (**B**), aspartate aminotransferase (AST) (**C**), triglycerides (TG) (**D**). Glucose (Glu) levels in mice fed for 12 weeks (**E**). Serum levels of low-density lipoprotein (LDL-C) (**F**), total cholesterol (TC) (**G**), and high-density lipoprotein (HDL-C) (**H**). Liver levels of glutathione peroxidase (GSH-PX) (**I**), malondialdehyde (MDA) (**J**), glutathione (GSH) (**K**), superoxide (SOD) (**L**), catalase (CAT) (**M**), and total antioxidant capacity (T-AOC) (**N**). Data are expressed as the mean ± SEM and were analyzed via one-way ANOVA. Asterisks (*, **, and ***) represent significant differences with *p* < 0.05, *p* < 0.01, and *p* < 0.001, respectively. Hematoxylin–eosin-stained liver tissue section (original magnification 400×) (**O**). Oil-red O staining of paraformaldehyde-fixed liver sections prepared from the four groups of mice (original magnification 400×) (**P**). Experimental treatments: (1) Normal Diet (CON); (2) High-Fat Diet (HFD); (3) High-Fat Diet + Type 2 Resistant Starch (RS2); (4) High-Fat Diet + Indole-3-Propionic Acid (IPA).

After 12 weeks of HFD feeding, mice in the HFD group were significantly fatter and had steatosis as opposed to those in the CON group (Figure 1A). IPA and RS2 ameliorated hepatic steatosis, with RS2 having a more prominent effect. Mice in the HFD, RS2, and IPA groups had significantly higher blood glucose levels compared to the CON group (*p* < 0.05). However, within the HFD, RS2, and IPA groups, those fed RS2 and IPA had lower fasting blood glucose levels than those fed HFD alone (Figure 1E). The pancreases of CON, IPA, and RS2 mice showed normal islets of Langerhans, with distinct beta cells at the center and alpha cells at the periphery (Figure S2A,B). After 12 weeks on an HFD, degeneration of the pancreatic vesicle cells was observed, with most of the vacuolated spaces being in the periacinar region. There were no significant differences in food and energy intake between the four groups (Figure S1H,I).

The biochemical indices of mice were measured after 6 and 12 weeks of intervention. The results at six weeks are shown in Figure S1A–F, while those at 12 weeks are shown in Figure 1B–F. Serum ALT, AST, HDL-C, TC, and LDL-C were significantly higher in the HFD, RS2, and IPA groups compared to those in CON mice (*p* < 0.05), with no significant difference among the three groups (Figure 1B–D,F–G). However, AST, ALT, TG, and LDL-C levels in the RS2 group were lower than in the HFD group, while HDL-C levels were higher (Figure 1D). These results showed that an HFD could aggravate liver injury, which RS2 and IPA alleviated to some extent, with the former being more effective.

We also evaluated oxidative stress-related indices in the mouse liver (Figure 1M,N). Compared to the HFD group, the total antioxidant capacity (T-AOC) (Figure 1N) in the CON, RS2, and IPA groups was significantly increased (*p* < 0.01), while GSH contents (Figure 1K) in the RS2 and IPA groups exhibited an upward trend. Compared with the HFD group, MDA (Figure 1J), CAT (Figure 1L), and SOD (Figure 1M) levels were lower in both the RS2 and IPA groups. As shown in Figure 1J, MDA content in the liver of HFD group mice was significantly higher than that of the CON group mice. RS2 and IPA decreased MDA levels in the mouse liver, with RS2 exhibiting greater antioxidant activity.

While CAT, GSH, MDA, CAT, and GSH-Px (Figure 1I) levels did not differ among the four groups, T-AOC in the HFD group was significantly lower than in the other groups. These results indicated that fat accumulation led to a reduction in antioxidant levels and increased lipid peroxidation in the livers of HFD mice. Supplementing the diet with RS2 and IPA reduced fat accumulation and enhanced T-AOC, which in turn protected the liver from lipid peroxidation damage to a certain extent.

HE staining of liver tissue revealed a clear liver plate in control mice, with normal liver lobules and round, plump, regularly arranged hepatocytes, in the absence of obvious pathological changes (Figure 1O). Mice in the HFD group had disordered hepatocyte arrangement, hepatocyte edema, local hepatocyte necrosis, liver steatosis, and a large number of lipid droplet vacuoles in the cytoplasm. As opposed to the HFD group, the hepatocytes of mice in the RS2 and IPA groups retained normal morphology, with some showing slight edema, and the number of fat droplet area being significantly reduced (Figure S1J). Oil Red O staining of liver tissue showed that, compared to the control group, the HFD, RS2, and IPA groups exhibited significantly increased lipid droplets and hepatic lipid accumulation. Meanwhile, lipid droplets and accumulation were significantly reduced in the RS2 group (Figure 1P). Overall, the protective effect of RS2 against liver steatosis was better than that of IPA. No noteworthy changes were observed in the ileum and colon tissues (Figure S2C,D).

**Figure 2:**
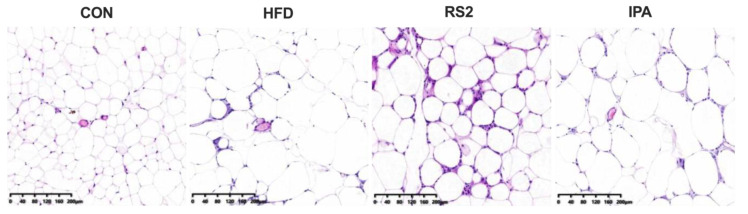
Hematoxylin–eosin-stained white adipose tissue sections (original magnification 200×) from CON, HFD, RS2, and IPA group mice. Experimental treatments: (1) Normal Diet (CON); (2) High-Fat Diet (HFD); (3) High-Fat Diet + Type 2 Resistant Starch (RS2); (4) High-Fat Diet + Indole-3-Propionic Acid (IPA).

In addition, HE staining results of adipose tissue revealed that, compared to the normal control group, white adipocytes were significantly increased in the HFD group, with obvious fat accumulation in adipose tissue. Compared to the HFD group, RS2 but not IPA feeding for 12 weeks reduced fat accumulation in adipocytes (Figure 2). These results indicated that RS2 rather than IPA improved abnormal fat metabolism in mice with HFD-induced obesity mice by reducing liver fat accumulation and adipocyte size in white adipose tissue.

### 3.2. Effects of RS2 and IPA on Serum Tryptophan Metabolites in Obese Mice

**Figure 3:**
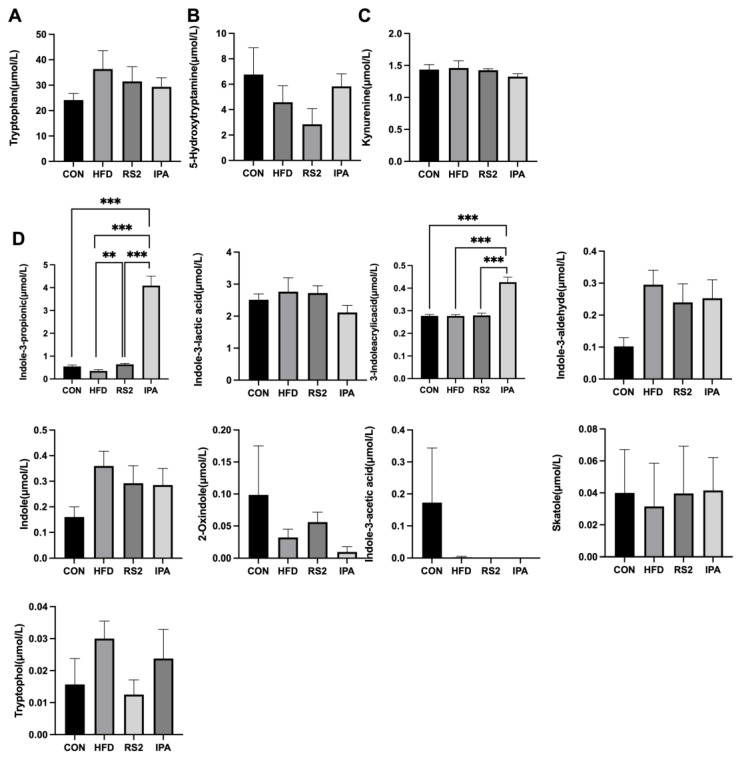
Serum tryptophan metabolite levels in the serum of mice. Tryptophan levels (**A**), 5-HT levels (**B**), kynurenine levels (**C**), changes in indole metabolite levels (**D**). Data are expressed as the mean ± SEM and were statistically analyzed via one-way ANOVA. Asterisks (**, and ***) represent significant differences with *p* < 0.01, and *p* < 0.001, respectively. Tryptophan (Trp), 5-hydroxytryptamine (5-HT), kynurenine (KYN), indole-3-propinic (IPA), indole-3-lactic acid (ILA), 3-indoleacrylicacid (IA), indole-3-aldehyde (IALD), indole-3-acetic acid (IAA). Experimental treatments: (1) Normal Diet (CON); (2) High-Fat Diet (HFD); (3) High-Fat Diet + Type 2 Resistant Starch (RS2); (4) High-Fat Diet + Indole-3-Propionic Acid (IPA).

Serum tryptophan metabolites levels were determined via LC-MS/MS. Compared to the other three groups, 3-indoleacrylicacid (IA) and IPA levels were significantly increased in the IPA group (*p* < 0.01) (Figure 3D). Compared to the HFD group, IPA levels in the RS2 group were significantly higher (*p* < 0.01).

### 3.3. Effects of RS2 and IPA on Gut Microbiota in Obese Mice

#### 3.3.1. Effect of RS2 and IPA on Alpha and Beta Bacterial Diversity

**Figure 4:**
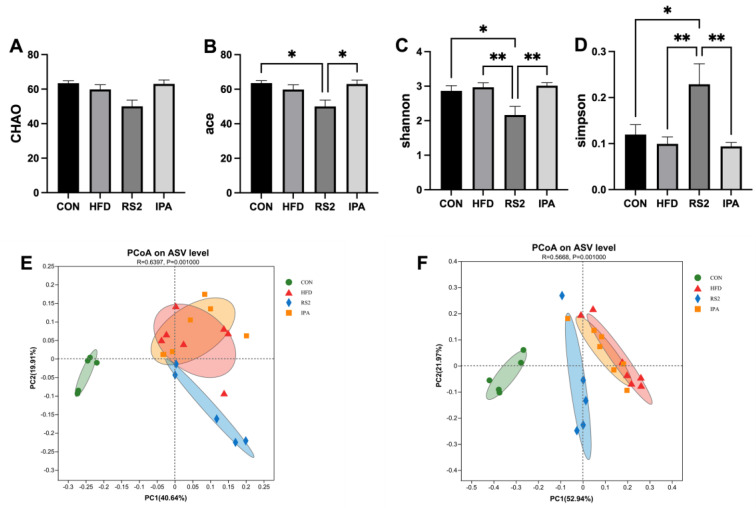
Effect of RS2 and IPA on alpha- and beta-diversity. Bacterial richness (Chao (**A**) and Ace (**B**) index) and diversity comparison (Shannon (**C**) and Simpson (**D**) index) between the four groups. Principal coordinate analysis (PCoA) based on unweighted UniFrac distances (**E**) and weighted UniFrac distances (**F**) of samples from the four groups. Asterisks (* and **) represent significant differences with *p* < 0.05 and *p* < 0.01, respectively. Experimental treatments: (1) Normal Diet (CON); (2) High-Fat Diet (HFD); (3) High-Fat Diet + Type 2 Resistant Starch (RS2); (4) High-Fat Diet + Indole-3-Propionic Acid (IPA).

To analyze changes in gut microbiota community structure in response to the RS2 and IPA feeding regimens, we performed 16S rRNA sequencing of cecal content collected from mice after 12 weeks of dietary intervention. We obtained 1,466,735 high-quality reads. The alpha and beta diversity of intestinal bacteria was compared between all samples. After 12 weeks of dietary intervention, the ace index of the HFD group showed a decreasing trend compared to the control group. In contrast, the ace index of the RS2 group was significantly lower than that of the control group (*p* < 0.05) (Figure 4A). Additionally, the ace index in the IPA group was significantly higher than in the RS2 group (*p* < 0.05). The Shannon value of the RS2 group was significantly lower, while the Simpson value was significantly higher when compared to those of the other three groups (*p* < 0.05) (Figure 4D). These results indicate that the HFD reduced community diversity, which could not be reversed by RS2 as opposed to IPA.

As for beta diversity, no clear visual separation between the HFD and IPA groups was observed in the plot of unweighted UniFrac-based distance principal coordinate analysis (PCoA) (Figure 4E). The weighted UniFrac distance (Figure 4F) also showed that there was obvious aggregation of bacterial composition for the HFD and IPA groups. However, the CON and RS2 groups showed a clear visual separation from the other two groups. The RS2 group was closer to the CON group, and the ANOSIM analysis based on unweighted UniFrac-based distance (R = 0.6397, *p* = 0.001) and weighted UniFrac distance (R = 0.5668, *p* = 0.001) indicated a significant difference in treatments. These results indicate that RS2 but not IPA treatments significantly affected the beta diversity of bacterial communities.

#### 3.3.2. Effect of RS2 and IPA on Composition of the Bacterial Community Composition

**Figure 5:**
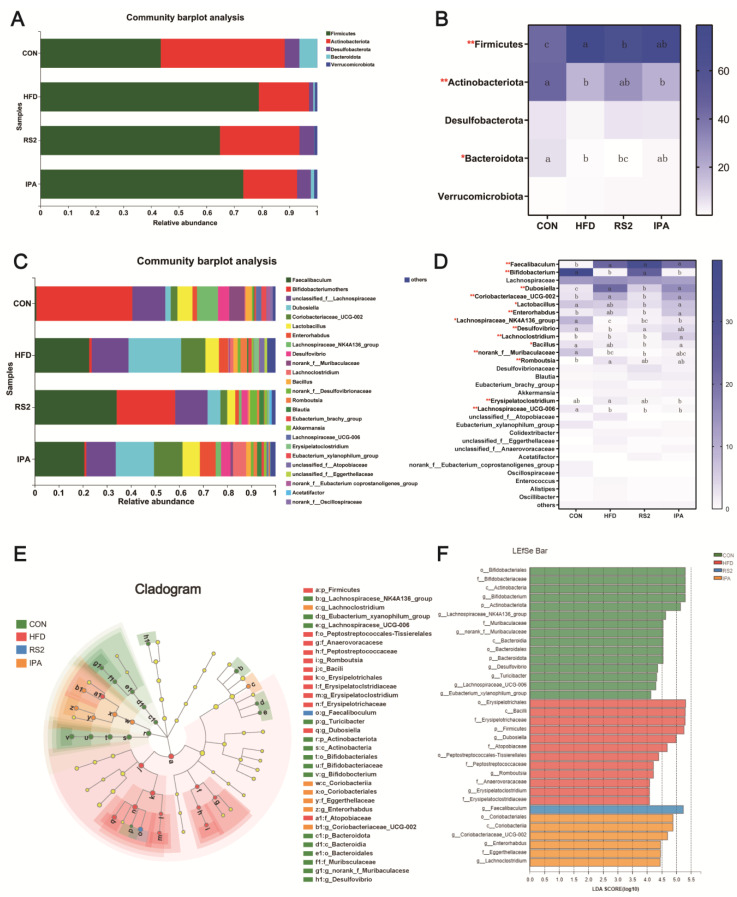
Relative abundance of bacterial phyla (**A**) and genera (**C**). Heat maps of the mean relative abundances of the prominent phyla (**B**) and genera (**D**). In B and D, mean values with different letters are significantly different (*p* ≤ 0.05). Significant correlations are marked by * *p* < 0.05 and ** *p* < 0.01. The cladogram of linear discriminant analysis (LDA) effect size (LEfSe) of microbial abundance from the phylum to genus level (**E**). LDA score assessments of difference between the four groups, with a score threshold of 4.0 (**F**). Experimental treatments: (1) Normal Diet (CON); (2) High-Fat Diet (HFD); (3) High-Fat Diet + Type 2 Resistant Starch (RS2); (4) High-Fat Diet + Indole-3-Propionic Acid (IPA).

Firmicutes, Actinobacteriota, Desulfobacterota, Bacteroidota, and Verrucomicrobiota were the most abundant phyla identified in the tested samples (Figure 5A). After HFD treatment, the abundance of Firmicutes increased remarkably from 43.37% to 78.90% (*p* < 0.01), whereas the abundance of *Actinobacteria* significantly decreased from 44.76% to 18.12% (*p* < 0.01). Following RS2 intervention, the abundance of *Firmicutes* significantly decreased from 78.90% to 64.87% (*p* < 0.05). The abundance of the *Actinobacteria* increased from 18.12% to 28.68%, without significance (Figure 5B).

The bacterial genera detected at ≥1% average relative abundance are shown in Figure 5C. Genus-level analysis revealed *Faecalibaculum, Bifidobacterium*, *unclassified_f_Lachnosporaceae*, and *Dubosiella* as the four most dominant genera in mouse cecal contents. At the genus level, *Bifidobacterium*, *Lachnospiraceae_NK4A136_group*, *Desulfovibrio*, *norank_f_Muribaculaceae*, and *Lachnospiraceae_UCG-006* were found to be more abundant in the CON group (38.86%, 8.71%, 4.64%, 6.40%, and 2.18%, respectively) than in the HFD group (1.03%, 0.17%, 0.38%, 0.29%, and 0.13%, respectively, *p* < 0.01). Following the RS2 intervention, there was a significant increase in the abundance of *Bifidobacterium* and *Desulfovibrio* compared to the HFD group, from 1.03% to 24.29% (*p* < 0.01) and from 0.38% to 2.41% (*p* < 0.05), respectively. Conversely, the abundances of *Dubosiella* and *Coriobacteriaceae_UCG-002* significantly decreased, from 21.87% to 5.35% and from 10.06% to 2.81% (both *p* < 0.01), respectively. *Lactobacillus, Bacillus, Enterorhabdus, Lachnoclostridium*, and *Erysipelatoclostridium* were also reduced, while *Lachnospiraceae_NK4A136_group* increased, yet without significance. After the IPA intervention, *Lachnospiraceae_NK4A136_group* and *Lachnoclostridium* abundance significantly increased compared with that in the HFD group (0.17% vs. 2.25%, 1.35% vs. 5.44%; *p* < 0.05), while the abundance of *Erysipelatoclostridium* remarkably decreased from 2.02% to 0.59% (*p* < 0.01) (Figure 5D).

The linear discriminant analysis (LDA) effect size (LEfSe) method was employed to detect bacterial taxa that exhibited significant differences in abundance among the CON, HFD, RS2, and IPA groups. A total of 34 bacterial clades presented statistically significant differences in abundance, with an LDA score of 4.0 (Figure 5F). The most differentially abundant bacterial taxa in the CON group belonged to *o_Bifidobacteriales*, *f_Bifidobacteriaceae*, *c_Actinobacteria*, and *g_Bifidobacterium*. The most differentially abundant bacterial taxa in the HFD group were *o_Erysipelotrichales*, *c_Bacilli*, *f_Erysipelotrichaceae*, and *p_Firmicutes*. The most differentially abundant bacterial taxa in the RS2 group belonged to *g_Faecalibaculum*. For the IPA group, the most differentially abundant bacterial taxa belonged to *o_Coriobacteriales* and *c_Coriobacteriia*.

### 3.4. The Relationship between Bacterial Community and Tryptophan Metabolism

**Figure 6:**
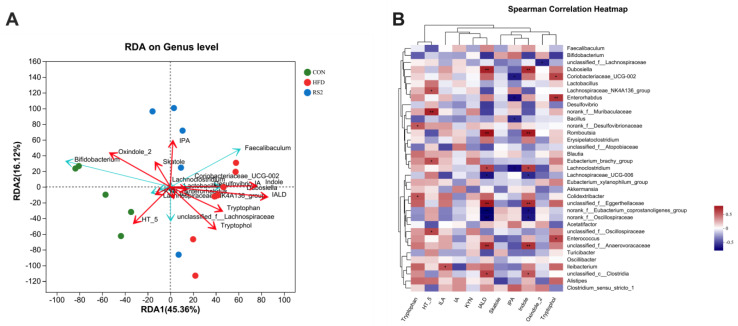
Correlation between microbial structure and tryptophan metabolism-related indices. Redundancy analysis (RDA) of the prominent phyla responding to tryptophan-related metabolites (**A**); A heatmap of Spearman’s correlation between the prominent genera and tryptophan-related metabolites (**B**). The intensity of the colors represents the degree of association (red, positive correlation; blue, negative correlation). Asterisks (* and **) represent significant differences with *p* < 0.05 and *p* < 0.01, respectively. 5-HT(HT-5), 2-oxindole (oxindole-2). Experimental treatments: (1) Normal Diet (CON); (2) High-Fat Diet + Type 2 Resistant Starch (RS2); (3) High-Fat Diet + Indole-3-Propionic Acid (IPA).

The association between dominant gut bacteria and tryptophan metabolism was assessed using RDA and Spearman’s correlation analyses. As expected, RS2 supplementation was positively correlated with IPA levels (Figure 6A). Enriched *Coriobacteriaceae_UCG-002* and *Enterorhabdus* in the IPA group were positively correlated with tryptophol levels. This relationship was confirmed by the significant positive correlations between tryptophan and *Coriobacteriaceae_UCG-002* (rS = 0.5541, *p* < 0.05), *Enterorhabdus* (rS = 0.6073, *p* < 0.05), and *Enterococcus* (rS = 0.547848108564, *p* < 0.05) (Figure 6A). Moreover, the *Lachnoclostridium* genera enriched in the IPA group were positively correlated with indole (rS = 0.5735, *p* < 0.05) and IALD (rS = 0.5613, *p* < 0.05). *Coriobacteriaceae_UCG-002* (rS = −0.5343, *p* < 0.05), *Enterorhabdus* (rS = −0.5711, *p* < 0.05), *Bacillus* (rS = −0.4902, *p* < 0.05), and *Lachnoclostridium* (rS = −0.5784, *p* < 0.05) were negatively correlated with IPA concentration, which had a strong positive correlation with *Clostridium_sensu_stricto_1* (rS = 0.4643, *p* = 0.0604) and *Bifidobacterium* (rS = 0.4093, *p* = 0.1028).

Functions of the bacterial community were predicted by PICRUSt2 (Figure S4). A total of 20 KEGG pathways (level 2) were screened, with relative abundances >0.5% presented in the four groups. Compared to the CON group, RS2 significantly promoted bacteria associated with amino acid metabolism, glycan biosynthesis, and metabolism, as well as cell Growth and death, while decreasing those implicated in lipid metabolism and infectious disease: bacterial. Compared to HFD, RS2 significantly increased bacteria implicated in cell growth and death, while decreasing bacteria associated with folding.

## 4. Discussion

RS2 has attracted significant interest in the field of human health, specifically in relation to combating and preventing obesity and diabetes [23,29]. However, the detailed mechanisms underlying its anti-obesity and anti-diabetic effects remain unclear. RS may prevent T2DM and obesity through various mechanisms, including the limitation of gluconeogenesis and the promotion of glycogenesis, promoting *Bifidobacterium* proliferation, promoting butyrate production, maintaining lipid homeostasis, and improving pancreatic dysfunction [30]. The present study aimed to explore whether RS2 can treat obesity through modulation of tryptophan metabolism, a novel mechanism underlying its prebiotic activity. We evaluated and compared the regulatory effects of RS2 and IPA on steatosis, serum tryptophan metabolites, and gut microbiota in obese mice. As expected, DIO mice fed with an HFD showed increased body weight and experienced liver injury. RS2 was more effective than IPA at reversing HFD-induced liver damage and gut dysbiosis.

Our results showed that RS2 intervention had no significant effect on weight loss or glycolipid metabolism in obese mice; stopped HFD mice weighed significantly less than HFD mice (Figure S1G). These results were in line with several rodent experiments reporting that RS could reduce body fat while having no effect on body weight [31,32]. Oral administration of RS2, while reducing the intake of an HFD, may have a more pronounced effect on weight loss. In addition, both the proportion of RS2 in the fodder and fodder type (high fat or not) can affect body weight. Body weight decreased only when the fodder contained 8% RS or more [33]. Additional research has indicated a potential correlation between the decrease in body weight observed during a 12-week RS2 intervention and the time delay necessary for preceding alterations in the gut microbiome [34]. One study confirmed that RS has a promising effect on glycemic control [29]. In humans, a decrease in blood glucose levels was observed after RS intake, although no change was observed in body weight or fat mass [35]. This is inconsistent with our results, wherein mice fed with RS2 showed no significant reduction in blood glucose levels. It is well known that body weight is closely related to the balance of caloric intake and expenditure, and therefore, it seems extremely important to determine and accurately analyze food and energy intake in mice. Future studies should further focus on this aspect in order to gain a more comprehensive understanding of the potential applications of RS2 and IPA in the treatment of obesity.

GSH levels and T-AOC are indicative of oxidative stress. Decreased T-AOC and GSH levels have been described in T2DM [36]. Lower GSH levels have also been reported in diabetes [37], senility [38], and cancer [39]. Interestingly, the RS2- and IPA-fed mice had higher circulating levels of GSH and T-AOC, suggesting that they may experience less oxidative stress in the liver, owing to the antioxidant effects of IPA. AST and ALT are primarily present in the cytosol of hepatocytes. When the liver is damaged, these two enzymes are released into the blood [40], so their serum levels reflect the degree of liver damage to a certain extent. The increased level of AST in response to HFD feeding was attenuated by RS2 and IPA treatments. Numerous studies have reported HFD-induced hepatic steatosis in mice [41,42]. Both RS2 and IPA alleviated hepatic steatosis in HFD-fed mice, with RS2 having a greater effect. Consistent with our results, Rosado et al. found that mice fed RS had reduced hepatic steatosis [43]. IPA may potentially mediate the positive impact of RS2 on liver health by inhibiting the production of intestinal endotoxins [44] as well as prevent oxidative stress and lipid peroxidation. Another study reported that IPA supplementation protected rats from HFD-induced NASH [45]. IPA suppresses NF-κB signaling and decreases the expression of proinflammatory cytokines, including TNFα, IL-1β, and IL-6, upon exposure to endotoxin, thereby alleviating hepatic inflammation and liver damage [44]. IPA can influence specific organs by circulating in the bloodstream. It establishes a connection between the gut and the liver, known as the gut–liver axis. This axis plays a crucial role in maintaining overall balance within the body and regulates the immune system. By doing so, it exhibits anti-inflammatory and antioxidant properties, working in harmony to modulate various physiological processes [46,47]. We hypothesized that protection of microbial IPA production is one of the mechanisms through which RS2 plays a prebiotic role. Additional research is required to investigate the impact and underlying mechanisms of IPA on host well-being and pathological conditions.

Gut microbes play key roles in regulating host metabolism [48]. In our study, dietary RS2 intervention altered the gut microbiota composition and reduced bacterial alpha diversity; this observation aligns with the results reported in Bendiks et al.’s study [43]. Although RS2 and IPA supplementation did not enhance α-diversity in obese mice fed an HFD, PCoA analysis using weighted UniFrac distances revealed that RS2 reduced gut dysbiosis caused by the HFD. This made the bacterial composition in the RS2 group more similar to that of the control group. HFD feeding increased the abundance of Firmicutes and decreased the abundance of *Bacteroidota* and *Actinobacteriota*. After RS2 intervention, Firmicutes abundance decreased, whereas *Actinobacteriota* abundance exhibited a trend toward increase. Several studies have proposed that the Firmicutes/Bacteroidetes ratio in the gut microbiota may be higher in obese animals and humans compared to normal-weight individuals, suggesting its potential as a biomarker [49]. However, our research indicates that it is challenging to link the Firmicutes/Bacteroidetes ratio to health status or view it as a definitive indicator of obesity [50].

At the genus level, the RS2 group exhibited a noteworthy elevation in *Faecalibaculum*, which made a substantial contribution to the overall variations in composition. *Faecalibaculum* species, such as *F. rodentium*, produce lactic acid as a major metabolite end product [51]. Due to the potential anti-obesity activity of lactic acid-producing bacteria, the observed rise in Faecalibaculum could potentially contribute to the favorable impact of RS2 [52,53]. RS2 supplementation also increased *Bifidobacterium* and *Desulfovibrio* abundance. *Bifidobacterium* is a well-known probiotic that confers health benefits [54]. Studies in humans have suggested an association between low levels of *Bifidobacterium* and obesity [55]. However, the association between *Bifidobacterium* and obesity is probably also species-specific [56]. A high-fat diet (HFD) is linked to a decrease in *Bifidobacterium*, a type of bacteria that has the ability to produce indoles from tryptophan [45,57]. Our findings further corroborate the notion that the inclusion of dietary RS plays a significant role in enhancing host well-being through the modulation of bacteria, such as Bifidobacterium, which are associated with improved gut health [58,59]. Hong et al. showed that *Desulfovibrio* can effectively reduce hepatic steatosis. This effect may occur because *Desulfovibrio* produces acetic acid and alters hepatic lipid metabolism in mice [60]. Ling et al. discovered that the *Coriobacteriaceae_UCG-002* is a potential harmful bacterium [61]. *Coriobacteriaceae_UCG-002* has been reported to promote intestinal cholesterol absorption and is positively correlated with liver TG levels [62]. We hypothesized that RS2 would reduce serum TG levels by reducing the levels of *Coriobacteriaceae_UCG-002*, thereby inhibiting cholesterol absorption. Notably, *Lachnospiraceae* NK4A136 group is a butyrate-producing bacterium [63], which was decreased in obese mice and subsequently increased by the IPA intervention. This group was previously reported to maintain gut barrier integrity in mice and is negatively correlated with intestinal permeability [64]. Our findings support RS2 restored gut dysbiosis to a greater extent than IPA.

There are three primary pathways through which ingested tryptophan can be metabolized: the production of serotonin, the production of kynurenine and its metabolites, or the transformation of indole and its derivatives [45]. Although microbial enzymes can only influence certain steps in the kynurenine pathway, the indole pathway relies entirely on microbial metabolism [65]. In the current study, we speculated that tryptophan metabolites may be important messengers that mediate microbe–host crosstalk. The interaction of the aryl hydrocarbon receptor (AhR) pathway with tryptophan metabolites is linked to energy metabolism and metabolic syndrome because the ability of tryptophan to metabolize into AhR binding derivatives is reduced in both preclinical and clinical metabolic syndromes [66]. Koay et al. found that RS2 can significantly increase the concentration of IPA in mouse plasma and is positively correlated with an increased abundance of *Allobaculum* and *Bifidobacterium* in the gut microbiota. Therefore, IPA may be a microbial metabolite that mediates RS2-induced health benefits [16]. It is important to highlight that a marked increase in serum IPA was also found after RS2 intervention in our study, which is consistent with the findings of Koay et al. In addition, IPA production was positively correlated with *Bifidobacterium* and *Clostridium*, suggesting that RS2 may stimulate gut microbes to produce IPA by promoting ILA-producing *Bifidobacterium* and IPA-producing *Clostridium* [67,68]. This hypothesis remains to be confirmed in future research.

Previous studies have revealed that IPA derived from dietary Trp via gut microbiota transformation is negatively correlated with T2DM and systemic low-grade inflammation [6,69]. Moreover, orally administration of low-dose (20 mg kg^−1^) IPA for just 4 days is effective in maintaining the integrity of the gut barrier in HFD-fed mice [70]. Therefore, the use of IPA shows promising potential in the diagnosis and treatment of metabolic disorders. Chen et al. found that the high-dose (100 mg kg^−1^) but not low-dose (30 mg kg^−1^) IPA could be a potential therapeutic against obesity [71]. They found that obesity leads to a dramatic decline in serum and colonic mucosa levels of IPA, with IPA supplementation exerting beneficial effects on weight and glycolipid metabolism disorders, which are not closely related to gut microbiota composition. Although we also confirmed that IPA had minor effects on the intestinal microbiome as opposed to RS2, we did not observe any weight loss effect of low-dose IPA (20 mg kg^−1^). This may be due to the long-term HFD feeding. An insufficient dose of IPA could be another reason. In the present study, prophylactic rather than therapeutic doses were used, indicating that the effectiveness of IPA as a therapeutic intervention relies on factors such as the dosage administered, the specific organ being targeted, and the timing of the treatment. The appropriate doses of IPA should be further explored in future studies to refine existing treatment options.

This study is subject to certain limitations. Although introducing IPA intervention subsequent to obesity induction indicated that low-dose IPA had no therapeutic effect on obese mice, we could not determine whether IPA had a preventive effect on obesity. Our subsequent research will examine the low-dose IPA’s preventive effect on obesity and the high-dose IPA’s therapeutic effect. We hypothesize that a combination of prebiotic (RS2) and potential postbiotic (IPA) treatment strategies could provide a range of theoretical advantages in combating obesity. Therefore, this combination should be considered as an additional intervention option, rather than simply a measure of RS2 and IPA efficacy for obesity treatment in isolation. Future research could explore the outcomes of combined prebiotic and postbiotic treatment for obesity.

## 5. Conclusions

Although RS2 and IPA did not reduce body weight or improve glycolipid metabolism in obese mice while the high-fat diet was maintained, our results suggest that the use of prebiotic RS2 or a potential postbiotic IPA intervention may make weight loss in the presence of cessation of a high-fat diet more significant. However, RS2 exhibited superior efficacy in mitigating obesity-induced steatosis and restoring gut microbiome homeostasis when compared to prophylactic doses of IPA. The beneficial effects of RS2 were not solely attributed to the stimulation of IPA production by gut microbes; rather, RS2’s unique orthogonal and hexagonal crystal nanostructure allowed for direct regulation of host sugar and fat metabolism. Future investigations should focus on exploring the underlying mechanisms through which RS2 promotes overall health from this perspective.

## Data Availability

The obtained sequence data were deposited in the NCBI Sequence Read Archive (SRA) database under the BioProject ID PRJNA935523. Other raw data supporting the conclusions of this paper will be provided by the authors.

## Supplementary Materials

The following supporting information can be downloaded at https://www.mdpi.com/article/10.3390/foods13111625/s1: Figure S1: Serum levels of alanine aminotransferase (ALT) (A), aspartate aminotransferase (AST) (B), total cholesterol (TC) (C), triglycerides (TG), low-density lipoprotein (LDL-C) (E) and high-density lip-oprotein (HDL-C) (F) were measured. Body weight of mice was recorded at 12 weeks(G). Average daily food intake and energy intake of mice were recorded (H and I). Fat droplet area in liver tissue section (J).; Figure S2: The pancreas in the haematoxylin-eosin stained tissue section (original magnification 200× (A), 400× (B)). The ileum (C) and colon (D) in the haematoxylin eosin stained tissue section (original magnification 200×); Figure S3: Venn analysis of shared ASV in four groups.; Figure S4: Comparison of microbial function prediction. PICRUSt2-predicted relative abundances of the KEGG pathway (KEGG level 2).
