# Supplementary material for: The Effect of Type 2 Resistant Starch and Indole-3-Propionic Acid on Ameliorating High-Fat-Diet-Induced Hepatic Steatosis and Gut Dysbiosis
Source: Foods. 2024 May 23;13(11):1625. doi: 10.3390/foods13111625 (PMC11172015; doi:10.3390/foods13111625)
Supplement: Supplementary file 1 [file foods-13-01625-s001.zip › foods-2998447-supplementary.pdf]

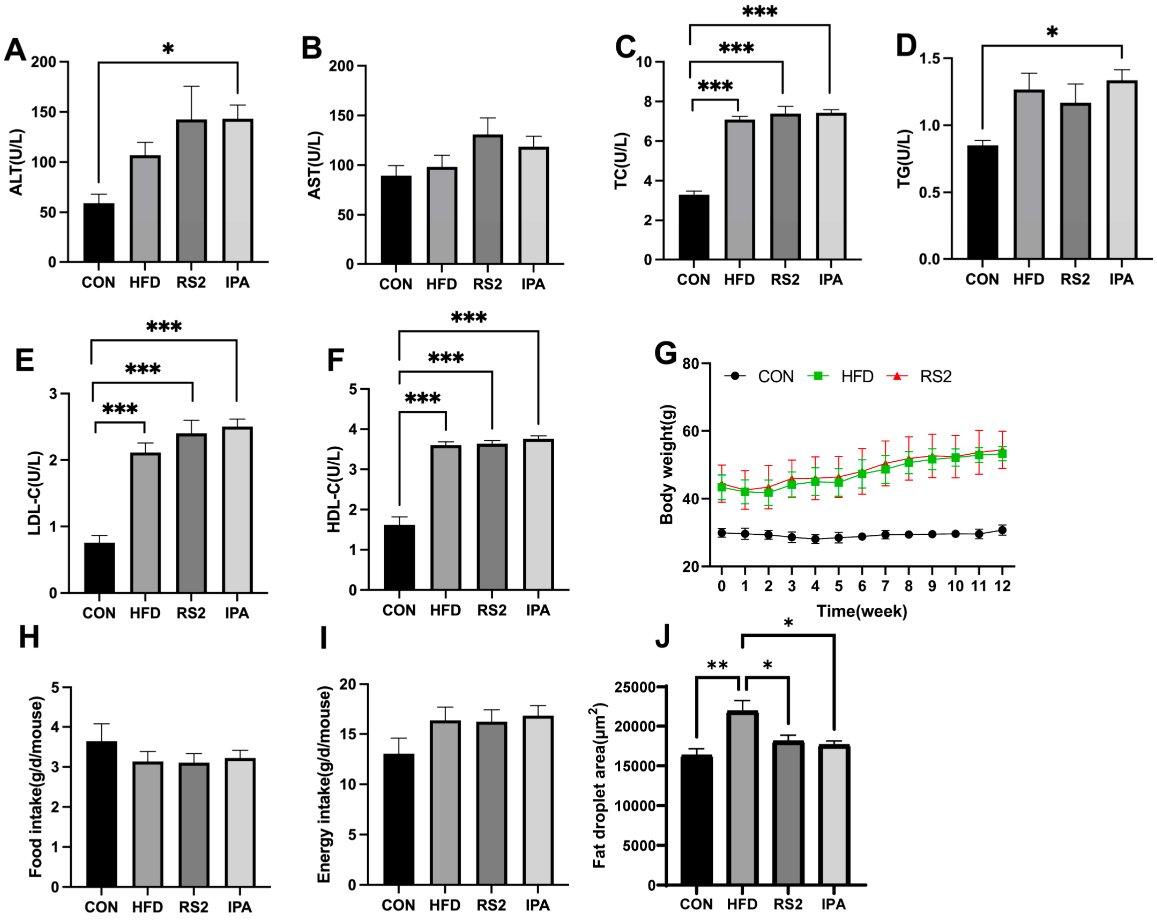

Figure S1. Serum levels of alanine aminotransferase (ALT) (A), aspartate aminotransferase (AST) (B), total cholesterol (TC) (C), triglycerides (TG), low-density lipoprotein (LDL-C) (E) and high-density lipoprotein (HDL-C) (F) were measured. Body weight of mice was recorded at 12 weeks(G). Average daily food intake and energy intake of mice were recorded (H and I). Fat droplet area in liver tissue section (J). Experimental treatments: 1) Normal Diet (CON); 2) High-Fat Diet (HFD); 3) High-Fat Diet + Type 2 Resistant Starch (RS2); 4) High-Fat Diet + Indole-3-Propionic Acid (IPA).

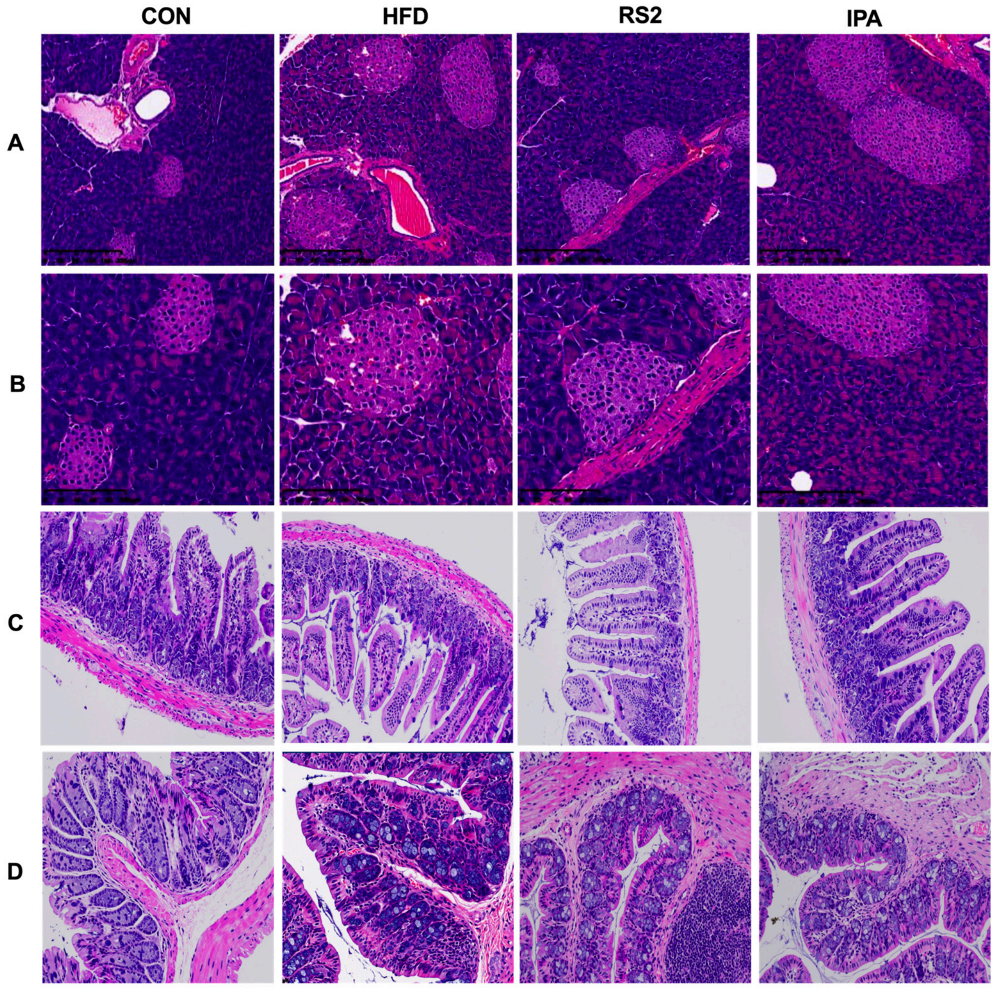

Figure S2: The pancreas in the haematoxylin-eosin stained tissue section (original magnification 200X(A)，400X(B)). The ileum (C) and colon (D) in the haematoxylin eosin stained tissue section (original magnification 200X). Experimental treatments: 1) Normal Diet (CON); 2) High-Fat Diet (HFD); 3) High-Fat Diet + Type 2 Resistant Starch (RS2); 4) High-Fat Diet + Indole-3-Propionic Acid (IPA).

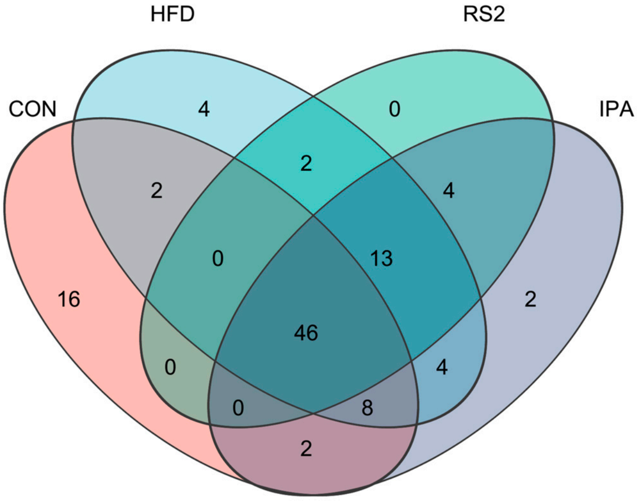

Figure S3: Venn analysis of shared ASV in four groups.

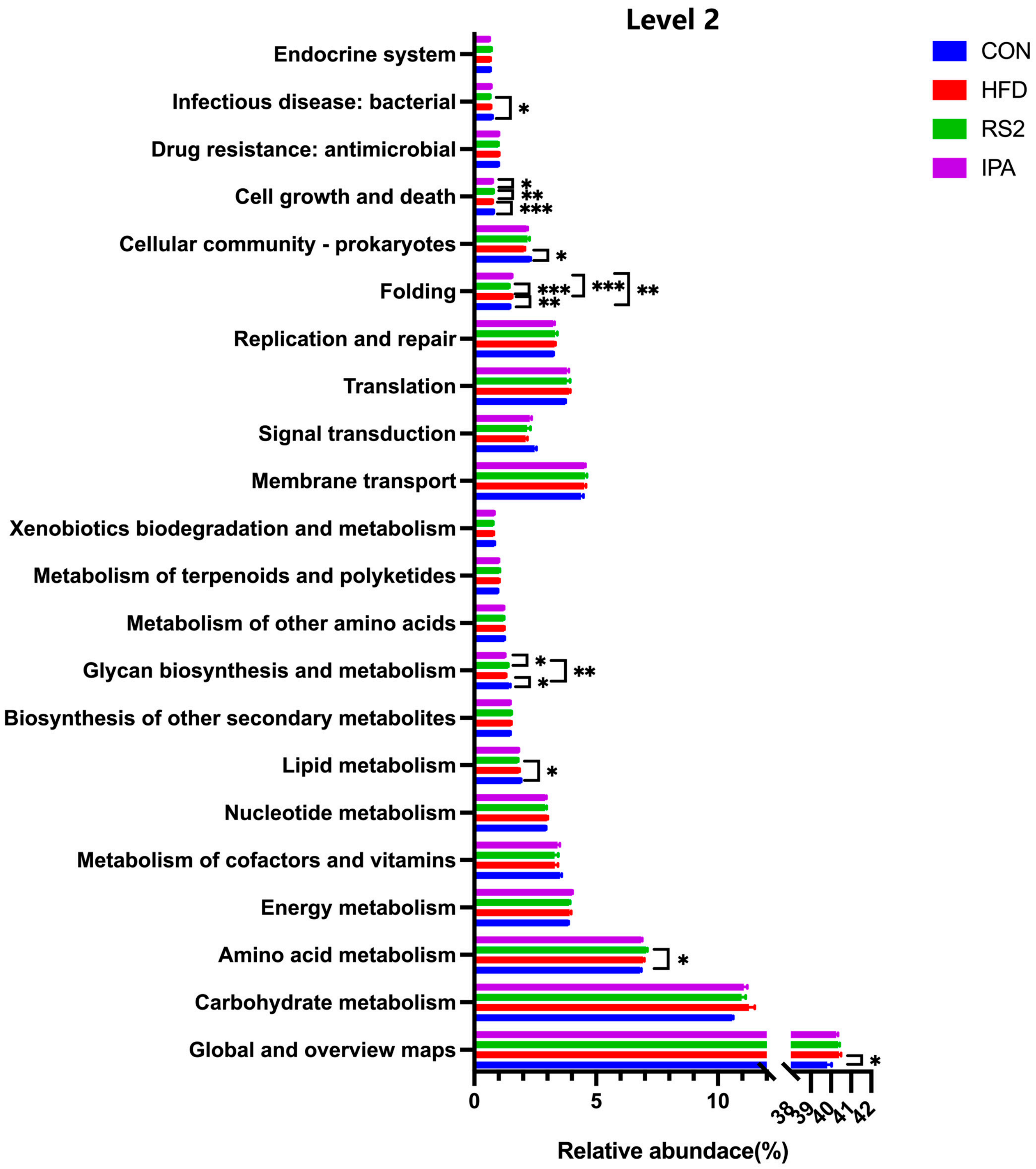

Figure S4: Comparison of microbial function prediction. PICRUSt2-predicted relative abundances of the KEGG pathway (KEGG level 2). Data were expressed as mean ± SEM and statistically analyzed by using one-way ANOVA; Asterisks (*, ** and **) represent significant differences with $P < 0.05$, $P < 0.01$ and $P < 0.001$, respectively. Experimental treatments: 1) Normal Diet (CON); 2) High-Fat Diet (HFD); 3) High-Fat Diet + Type 2 Resistant Starch (RS2); 4) High-Fat Diet + Indole-3-Propionic Acid (IPA).
